# Williams syndrome: on the role of intellectual abilities in anxiety

**DOI:** 10.1186/s13023-021-02098-4

**Published:** 2021-11-07

**Authors:** Charlotte Willfors, Deborah M. Riby, Marcus van der Poll, Katja Ekholm, Hanna Avdic Björlin, Johan Lundin Kleberg, Ann Nordgren

**Affiliations:** 1 Rare Diseases Research Group, Department of Molecular Medicine and Surgery, Karolinska Institutet, BioClinicum, J10:20, Visionsgatan 4, 171 64 Stockholm, Sweden; 2 Department of Clinical Genetics, Karolinska University Laboratory, Karolinska University Hospital Solna L5:03, 171 64 Stockholm, Sweden; 3 Department of Psychology, Durham University, South Road, Durham, DH1 3LE UK; 4 Centre for Psychiatry Research, Department of Clinical Neuroscience, Karolinska Institutet, Stockholm Health Care Services, Gävlegatan 22, 113 30 Stockholm, Sweden

## Abstract

**Background:**

Individuals with Williams syndrome (WS) have an elevated risk for anxiety disorders throughout the life span, making it a research priority to identify the individual factors associated with anxiety. Most of the existing literature is based on questionnaire data and suggests that impaired executive functions (EF) increase the risk for anxiety in WS. The aim of this study was to use direct measures by trained clinicians to investigate the effects of general intelligence, inhibition, sustained attention, and working memory on anxiety in WS, to further elucidate potential underlying mechanisms.

**Method:**

Twenty-four individuals with WS participated in the study (mean age: 29 years, range: 9–53 years), together with at least one of their parents. The MINI international neuropsychiatric interview for DSM-5 was completed to establish clinical diagnosis of anxiety, and the Clinical Global Impression Scale – Severity was used for an expert rating of symptom severity. Intellectual abilities were measured using the Wechsler scales, and attention and inhibition using the Conner’s Continuous Performance Test. In addition, a parent-report questionnaire measuring EF, learning and memory was collected.

**Results:**

In contrast to the apriori hypothesis, there was no significant association between anxiety and core elements of EF such as working memory, sustained attention, and inhibition (i.e. the process of restraining one’s impulses or behaviour). Using ordinal logistic regression analyses, we showed that decreasing intelligence quotient (IQ) and age are associated with elevated anxiety. We confirmed these results in between-groups analyses (anxiety disorder vs no current anxiety disorder), and low IQ was associated with higher risk of having an anxiety diagnosis. In addition, Bayesian statistics gave substantial evidence for no significant association between anxiety and inhibition.

**Conclusion:**

By using direct measures of psychological pathology and functioning, the current results provide a deeper characterisation of the WS phenotype and provide novel insights into the potential mechanisms underpinning anxiety.

## Background

Williams syndrome (WS) is a developmental condition caused by a deletion of 26–28 genes on one copy of chromosome 7q11.23. It is a relatively rare condition with an estimated prevalence of 1 in 7500 live births [1]. The WS phenotype is characterised by distinct medical, cognitive, and behavioural features. The majority of individuals with WS have a mild to moderate intellectual disability, with relative strengths in verbal ability, and relative impairments in visuo-spatial and executive functions (EF) [2]. The reported EF difficulties associated with WS include impairment in inhibition, working memory and attentional problems in both verbal and visual spatial modalities [3–5]. The co-occurrence with attention-deficits/hyperactivity disorder (ADHD) in WS is high; > 60% in children, and 20% in adolescents [6]. However, difficulties with distractibility is reported in up to 85% of adults with WS [7]. At a non-clinical level, behavioural and neuropsychological features are proposed to be shared between WS and ADHD, for example in areas such as working memory strategies and delayed short-term memory [8].

Behaviourally, although there are vast individual differences, individuals with WS are described as hyper social, loquacious, empathic, and friendly. Still, the social phenotype in WS is complex, and the out-going and socially fearless personality is combined with autistic traits, including hypersensitivity, restricted and repetitive interests and behaviours, and social communication atypicalities [9], as well as difficulties in making and keeping friends [10, 11]. Longitudinal data however, suggest that the social functioning, ability to make friends and the quality of friendship, improves with time [12].

Individuals with intellectual disability are at increased risk for mental health disorders in comparison to the general population, and in WS anxiety disorders are particularly common [13]. Adults with WS have a four times higher risk of developing an anxiety disorder than other groups with intellectual disability [14]. Longitudinal studies suggest that anxiety in WS can be chronic and worsen over time without intervention [15, 16]. Notably, there is a large variability within the WS group regarding social motivation and autistic traits, as well as anxiety.

Deletions of the WS locus have been associated with altered functioning in several neuronal systems [2, 17]. In particular, the neuronal deletion of the General transcription factor II-I gene (*Gtf2i)* has been shown to cause myelination deficits, which have been linked to behavioural atypicalities such as motor deficits, increased sociability, and anxiety, in mice [18, 19]. In humans with WS, structural and functional brain alterations have been identified, and increased amygdala activity has been linked to the abnormal fear processing [17, 20]. Functional studies of the brain have shown that individuals with WS displayed elevated amygdala activity and abnormal connectivity between the amygdala and the orbitofrontal cortex in comparison to controls, when viewing threatening non-social stimuli [21]. Using diffusion tensor imaging, Avery and colleagues reported lower white matter integrity in prefrontal cortex-amygdala pathways in WS individuals in comparison to controls. The prefrontal cortex has a function of inhibiting amygdala activity, and therefore white matter deficits in the prefrontal cortex might contribute to hyperactivity in the amygdala. Hence, the deficits in the structural integrity of prefrontal–amygdala white matter pathways associated with WS, are proposed to underlie the increased amygdala activity and elevated levels of anxiety [17].

On a behavioural level, there are several potential mechanisms that have been proposed to be driving the heightened susceptibility to anxiety in WS [22]. Pitts and colleagues used logistic regression modelling in a large multi-cohort sample of WS individuals to examine how gender, age, intelligence quotient (IQ), and behaviour regulation affect the risk of receiving a specific phobia diagnosis [23]. They found that the probability for a diagnosis decreased with increased age and higher IQ. However, the strongest relationship was found for behaviour regulation difficulties; the probability of receiving a specific phobia diagnosis increased as behaviour regulation difficulties increased. Interestingly, McGrath and colleagues showed that anxiety levels, as well as verbal IQ, are associated with attentional bias to emotional faces in WS. That is, individuals with high verbal IQ and high levels of anxiety had an attentional bias to angry faces, whilst individuals with low verbal IQ and low levels of anxiety had a bias to happy faces [24]. In this study, no significant correlation between anxiety and IQ was found, and the authors suggest it might be due to power issues. However, other studies have also reported no association between IQ and anxiety in WS [16, 25, 26].

Ng-Cordell and colleagues examined the relationship between parent-reported anxiety, impairments in EF, and social ability. They found a relationship between anxiety and EF impairments, as well as between anxiety and social ability. Regression analyses revealed a strong relationship between high anxiety and low cognitive flexibility, and that this relationship might drive the association between anxiety and social functioning [15]. This study therefore proposes a link between anxiety and components of EF within the cognitive profile of WS.

Another mechanism that has been proposed to underpin anxiety is atypical sensory processing. Atypical sensory processing is common in WS, in particular hyperacusis that is reported in over 80% of children with WS [27, 28]. In a WS sample of mixed ages, the relationships between anxiety, hypersensitivity, and intolerance of uncertainty were examined. Both factors were shown to be independent predictors of anxiety, however intolerance of uncertainty played the most dominant role [29]. These results were replicated in a recent study, suggesting that the relationship between social profiles and anxiety is fully mediated by intolerance of uncertainty [30].

As pointed out in a meta review by Royston an colleagues, studies to date have been important in identifying potential mechanisms underlying anxiety in WS, however data are not based on direct measures of psychological functioning, and the direction of causality for the associations is still unclear [22]. In the study by South and colleagues [30], an elegant way to test the direction of causality is demonstrated through a mediation analysis. Still, the inclusion of direct measures of formal clinical evaluations, as opposed to research using parent report insights on non-clinical measures (e.g.[16, 25]), are scarce, and this is an important research priority to ensure that anxiety profiles are accurately captured, beyond parental reporting on questionnaires.

As well as direct clinical measures of anxiety, there is a need of more direct assessments of cognitive ability. Sustained attention and inhibition are proposed to be fundamental in supporting higher order cognitive functioning, and difficulties in these areas are associated with behavioural and emotional challenges [31]. Research on WS adults has reported inhibition deficits and problems re-engaging attentional control processes after making an error, as well as generalised attentional impairments [4]. Shalev and colleagues, used a novel method of analysis and examined the change in performance over time on a continuous performance test in a sample of WS and control groups [32]. They reported that individuals with WS performed worse over time, whilst the performance in the control groups were constant over time. The authors suggested that attention variability in particular, might be a sensitive measure to identify syndrome-specific behavioural markers in WS.

The current study will add to the literature on anxiety in WS, by combining clinical assessments with direct data from psychological tests and parental reports, thereby benefitting from a truly multi-methods and multi-informer approach to the question. The aim of the present study was to examine the associations between direct measures of attention, inhibition, working memory, IQ, and anxiety in WS. A second aim was to examine the associations between parent report of executive functions, learning and memory, and anxiety. Based on the existing literature, we hypothesized that more severe cognitive and executive functioning impairments (i.e. attention, inhibition, working memory, IQ) would be associated with higher levels of anxiety. In addition, we explored the effect of age on anxiety severity.

## Methods

### Participants

Individuals with WS (n = 24) were recruited from all over Sweden via patient organizations, and health care facilities. Inclusion criteria was a genetically confirmed WS diagnosis and that the individual was of age 6 years or older (no upper age limit). An equal number of males and females were included, and the age ranged from 9 to 53 years (mean = 29.4, SD = 13.4). WS diagnosis was confirmed through genetic examinations and/or medical records. Since many individuals were diagnosed early by Fluorescent in situ hybridization (FISH), there was no information about the size of the deletion available in most adults. The participants, and at least one of their parents, attended a two-day examination at the Karolinska University Hospital, including clinical interviews, neuropsychological testing, and biological sampling. A clinical psychologist and/or psychiatrist performed the diagnostic assessments, and a psychologist the neuropsychological testing. Based on the clinical assessment (see *Anxiety*), 15 out of 24 individuals (62.5% of the sample) were categorized as having one or more anxiety disorders (see Table 1).

**Table 1:** Prevalence of co-occuring psychiatric and neurodevelopmental disorders

Psychiatric/neurodevelopmental diagnoses	N	Percentage (%)
Anxiety disorders	15	62.5
Specific phobia	8	33.0
Panic disorder	3	12.5
Generalized anxiety disorder	3	12.5
Anxiety disorder not otherwise specified	2	8.0
Obsessive compulsive disorder	1	4.0
Post traumatic stress disorder	1	4.0
Intellectual disability	22	91.7
Mild	17	70.8
Moderate	5	20.8
ADHD	4	16.7
Autism spectrum disorder	3	12.5
Major depression disorder	3	12.5
Psychotic disorder	2	8.0
Tic disorder	1	4.0
Symptoms of oversensitivity	22	91.7

### Instruments

#### Anxiety

For a multi-informer approach, the assessment of anxiety was primarily based on self-assessment and expert ratings, and secondary on parent reports. Further, the aim was to have an exploratory approach and include a comprehensive psychiatric assessment, not only focusing on anxiety. Therefore the MINI or MINI KID international neuropsychiatric interview for Diagnostic and Statistical Manual of Mental Disorders (DSM-5) in combination with the Clinical Global Impression Scale – Severity (CGI-S) were used for assessment of anxiety. The MINI was completed with the individual and the parent, or the parent only (for younger children, n = 4), to establish clinical diagnoses of anxiety as well as other psychiatric disorders [33]. The assessors based their judgement on information from both the individual with WS and the parent(s), when both sources of information were available. In addition, an expert rating of the severity of anxiety symptoms was done using the CGI-S [34]. It is a seven-point scale ranging from “1 = normal, not at all ill” to “7 = among the most extremely ill patients”. The CGI-S has shown to have good validity and correlation with other clinical anxiety ratings as well as self-rated measures of anxiety severity [35]. Participants meeting the criteria for an anxiety diagnosis according to DSM-5 criteria, and having a CGI-S score of 3 or above, were assigned to the anxiety group. If a participant had more than one anxiety disorder, the most severe diagnosis (i.e. the one with the highest CGI-S score) was entered into the analyses. The clinical assessment of anxiety therefore resulted in a binary diagnostic classification as well as a dimensional severity rating.

#### General intelligence

General intelligence was assessed with the Wechsler scales. The Swedish version of Wechsler Intelligence Scale for Children 5th (WISC-V) was used for participants up to 16 years old (n = 4), and Wechsler Adult Intelligence Scale 4th edition (WAIS-IV) for participants older than 16 years (n = 20) [36, 37]. The 10 core subtests were administrated and converted to standard full scale IQ (FSIQ) score, verbal IQ (VIQ), performance/non-verbal IQ (PIQ; the Perceptual Reasoning index in WAIS-IV, and on the Fluid Reasoning Index in WISC-V), and working memory index (WMI) scores.

#### EF, memory and learning

For a multi-informer design, and to add broader definitions of executive functions and related areas important for every-day-life functioning, the Five to Fifteen (FTF) questionnaire was collected from the participants’ parents. The FTF was originally developed to assess ADHD and related problems in children aged 5 to 15 [38]. It comprises 181 items, which are rated on a 3-point Likert scale; “does not apply” (0), “applies sometimes or to some extent” (1), or “definitely applies” (2). The items are arranged in eight domains including EF, memory, and learning (the remaining five domains are not included in this study). The psychometric properties of the questionnaire have shown to be satisfactory. The internal consistency of the domains ranged from acceptable to very good. The FTF has a good test–retest reliability, and positive correlation with other scales such as the Child Behaviour Checklist [39]. Since there are no normative data for adults, we report the mean raw scores (range 0–2), and a higher score indicates more impairments. Cronbach’s alphas in the current sample were calculated and showed good to excellent internal reliability for the EF scale which consists of 25 items (α = 0.92), the Memory scale which consists of 11 items (α = 0.73), and the Learning scale which consists of 29 items (α = 0.94).

#### Sustained attention and inhibition

The Conners’s Continuous Performance Test (CCPT, [40]) is commonly used to assess attention characteristics of individuals with ADHD. Differing from more traditional continuous performance tests, the CCPT has a high signal to noise ratio, i.e. the subject is asked to respond to most stimuli, and the stimuli presentation rate varies between 1 and 4 s. Psychometric evaluations of the test show mixed results. The internal consistency is strong, but the test–retest reliability vary from adequate for commission errors and response time, to poor for omission errors [41]. In a factor analysis of the CCPT, the authors conclude that focusing on variability of commissions over time in CCPT, might be a unique measure of the test which at the time was still unexplored [42]. In the current study, the computerised child (K-CPT-2) or adult (CPT-3) versions of CCPT were used to assess attentional and inhibition abilities [40, 43]. To assess overall performance, we used correct responses and commission errors, in percentage. To assess change over time we used within-respondent variability (the amount of variability over 18 sub-blocks in relation to the standard deviation of the reaction time). A higher score represents a larger change in reaction times over time. Since no Swedish norms exist for the CCPT, raw scores were used in the analyses. A preliminary analysis showed no significant correlation between age and CCPT scores (CCPT CR r(22) = 0.123, *p* = 0.566; CCPT CE r(22) = 0.095, *p* = 658; CCPT Variability r(22) = 0.021, *p* = 0.928), and therefore, we did not control for age in the analyses including these variables.

#### Working memory

Working memory was assessed using the WMI from the Wechsler scales (see above for more details on the Wechsler scales). The WMI consists of two subscales, one based on auditory information, and one based on visual information. The WMI is calculated from both the verbal and visual subscales, and is converted to standard index score (mean = 100, SD = 15) and a higher score indicates better performance.

#### Hyper-sensory processing

For descriptive purpose, hypersensitivity was estimated from two questions (oversensitivity to touch, clothes etc., and oversensitivity to sound). The items were rated yes or no by the parents. A positive answer to at least one of the items gives a positive score of hypersensitivity.

### Data analysis

Descriptive analyses were used to characterize the sample. We used Pearson’s correlation coefficient to calculate the correlations between severity of anxiety (CGI-S scores), the different cognitive variables, and age. Due to directional hypotheses, one-tailed p-values are reported, except for age. Next, we did ordinal regression analyses to examine the relative contribution of related variables in predicting levels of anxiety. Variables significantly correlated with the CGI-S scores were entered into the model. Second, we did group comparisons to determine if there were differences between the two groups (anxiety disorder vs no anxiety disorder), in respect to the different outcome measures. Normality of the distribution and variance homogeneity were verified using Shapiro–Wilks test and Levene’s test, respectively. If the criteria were met, Student’s t test was used, and otherwise Mann–Whitney U test. Again, due to the directed hypothesis, one-tailed p-values are reported. Due to the unequal sample sizes, additional analyses with Welch’s t-test were performed, but did not change the significance of the results [12].

Since we found no significant associations between anxiety and several variables of interest, we applied Bayesian statistics to further explore the null hypothesis. A non-significant result in a traditional statistical analysis based on p-values can never be interpreted as reflecting the degree of support for the null hypothesis, but only as indicating that the null hypothesis could not be rejected and that the results are therefore non-conclusive. In contrast, Bayesian statistics quantify the relative likelihood of the null and the alternative hypothesis, and can therefore generate proof for the null hypothesis. Bayes factors (BF_10_) were calculated from the Bayesian Information Criteria, which quantifies the relative probability of the hypothesis (the model including the effect of interest) and the null (the same model without this effect). By convention, a BF_10_ > 3 indicates positive evidence for the hypothesis, a BF_10_ > 20 indicates strong support, and a BF_10_ > 150 very strong support [44]. By reversing the terms, a BF_01_ can be calculated, where larger numbers indicate higher probability of the null. The main strengths of Bayesian as compared to frequentist statistics is that it is less vulnerable to type I errors, and that it allows conclusions that the null hypothesis fits the data best. Uninformative priors were set for the Bayesian calculations and the JASP default settings were used [45, 46].

No corrections for multiple comparisons were applied due to the small sample size. All analyses were conducted using SPSS version 25 [47], except the Bayesian statistics, which were computed in JASP [45]. Study data were collected and managed using REDCap electronic data capture tools hosted at Karolinska Institutet [48, 49].

## Results

### Descriptive statistics

Out of 24 individuals, 15 had at least one anxiety disorders at the time of the assessment, including Specific phobia, Panic disorder, Generalized anxiety disorder, Anxiety disorder not otherwise specified, Obsessive compulsive disorder, and Post traumatic stress disorder, see Table 1. Additionally, three individuals had anxiety disorders that were at the time of assessment in partial remission. Of the specific phobias, the most common were phobias related to loud noises such as balloons, crying babies, and hand dryers. In addition the following types of phobias were reported; to sleep alone, to walk on uneven terrain, sharp objects, insects, and disorders related to the stomach. Other current neurodevelopmental and psychiatric disorders in the sample were Intellectual disability, ADHD, Autism spectrum disorder, Major depression, Psychotic disorders, and Tic disorder). The FSIQ in the sample ranged from 40 to 79 with an average of 57 (SD = 9.97). Symptoms of oversensitivity were reported in 22 of participants.

**Fig. 1:**
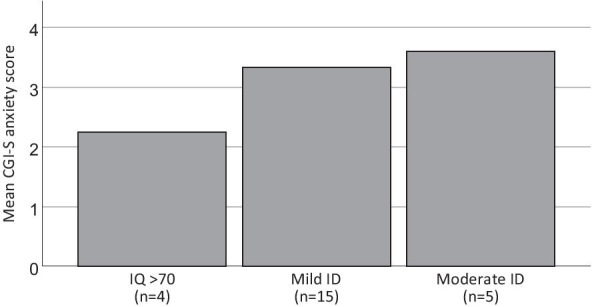
CGI-S anxiety scores split on intellectual disability severity groups. IQ = intelligence quotient, CGI-S = Clinical Global Impression Scale – Severity, ID = Intellectual disability

See Fig. 1 for CGI-S anxiety scores split on groups based on FSIQ; i) IQ > 70, ii) mild intellectual disability; IQ range between 50 and 69, and iii) moderate intellectual disability; IQ range between 36 and 49.

On a group level the CCPT commission errors and variability scores were “very elevated” in relation to an American normative sample (*t* score > 70) [40]. The FTF mean scores were compared to Danish norms (n = 3253) for ages 8–17 years [50]. The FTF domain scores included in the current study (i.e. EF, Learning, Memory), were all significantly elevated, i.e. there were more challenges reported in the WS group in relations to normative values (*p* < 0.001). Sex differences were examined and there were no between-sex differences in any variable.

### Correlations analyses

Correlations between anxiety severity and cognitive measures scores are shown in Table 2. These analyses showed that CGI-S anxiety scores were negatively correlated with FSIQ and verbal IQ, (therefore individuals with higher anxiety tended to have lower IQ and lower verbal ability). In addition, there was a negative association between CGI-S anxiety score and age, r(22) = **− **0.420, *p* = 0.040, i.e. younger individuals showed more severe anxiety symptoms. The statistical significance of the correlations did not change when using non-parametric tests. Bayes factors in favor of the null hypothesis for correlations between anxiety severity scores and cognitive/EF variables are also shown in Table 1. The Bayesian statistics gave positive evidence for the null hypothesis for FTF Learning scale (BF_01_ = 5.35, i.e. the data is 5.35 times more likely to occur under the null than the alternative hypothesis), and CCPT Commission error scores (BF_01_ = 5.22, i.e. the data is 5.22 times more likely to occur under the null than the alternative hypothesis). There is only weak evidence (BF_01_ < 3) for the null hypothesis for Working memory, FTF EF, FTF Memory, CCPT Correct responses and CCPT Variability, meaning that the results should be interpreted as inconclusive.

**Table 2:** Correlations among variables of interest, and Bayes factors for correlation with CGI-S anxiety

	CGI-S Anxiety	BF_01_ for CGI-S anxiety	FSIQ	PIQ	VIQ	WMI	FTF EF	FTF Memory	FTF Learning	CPT CR	CPT CE
FSIQ	− .365*	*0.49*	1								
Non-verbal IQ	**− **.327	*0.67*	.793**	1							
Verbal IQ	− .391*	*0.38*	.865**	.586**	1						
WM	**− **.105	*2.60*	.839**	.620**	.713**	1					
FTF EF	**− **.022	*2.65*	**− **.143	.121	**− **.261	.062	1				
FTF Memory	.109	*2.54*	**− **.111	.073	− .363*	.031	.683**	1			
FTF Learning	**− **.094	*5.35*	**− **.318	**− **.185	− .462*	**− **.135	.782**	.725**	1		
CCPT CR	**− **.093	*2.73*	.558**	.198	.506**	.544**	**− **.221	**− **.118	.229	1	
CCPT CE	.086	*5.22*	.358*	.008	.354*	.408*	**− **.222	**− **.246	**− **.169	.692**	1
CCPT Variability	.182	*1.78*	− .514**	− .481*	− .549**	**− **.303	.279	.057	.405*	− .409*	.175

Note: Significant correlations between variables are marked in bold. Bayes factors are marked in italic. CGI-S = Clinical Global Impression Scale – Severity, BF_01_ = Bayes Factor for the null hypothesis, FSIQ = Full scale IQ, PIQ = Performance/Non-verbal IQ, VIQ = Verbal IQ, WMI = Working Memory Index, FTF EF = Five to Fifteen, EF = Executive Function, CCPT = Connors Continuous Performance Test, CR = Correct Responses, CE = Commission Errors

*p < 0.05, **p < .001

### Regression analyses

Since FSIQ and age were significantly correlated with anxiety severity, these variables were entered as independent into the regression model, and CGI-S anxiety score as the dependent variable. A cumulative odds ordinal logistic regression with proportional odds was conducted. First, a full likelihood ratio test comparing the fitted model to a model with varying location parameters was used, which showed there was proportional odds, χ^2^(8) = 10.008, *p* = 0.264. Next, the deviance goodness of fit test showed that the model was a good fit to the observed data, χ^2^(113) = 76.251, *p* = 0.997. Although, there were zero frequencies in 83.3% of cells, the final model predicted the dependent variable, with statistical significance, over and above the intercept-only model, χ^2^(2) = 6.650, *p* = 0.036, accounting for 25% of the total variance in CGI-S anxiety score. The estimated odds ratios of increase in CGI-S anxiety score were not significant; 0.937 (95% CI, 0.866 to 1.013), *p* = 0.101 for increasing IQ (expressed as IQ points), and 0.949 (95% CI, 0.895 to 1.007), *p* = 0.082 for increasing age (expressed as years). In summary, IQ and age had a significant collective effect on anxiety with lower IQ and lower age being associated with more severe anxiety.

### Group comparisons

Next, the sample was split in two groups, individuals with an anxiety disorder (n = 15, 53% females, age; M = 27.6, SD = 14.1, range 9–53 years), and individuals with no current anxiety disorders (n = 9, 44% females, age; M = 32.3, SD = 12.5, range 16–49). Oversensitivity was reported in 93% of the participants in the anxiety group and 89% in the no anxiety group.

**Fig. 2:**
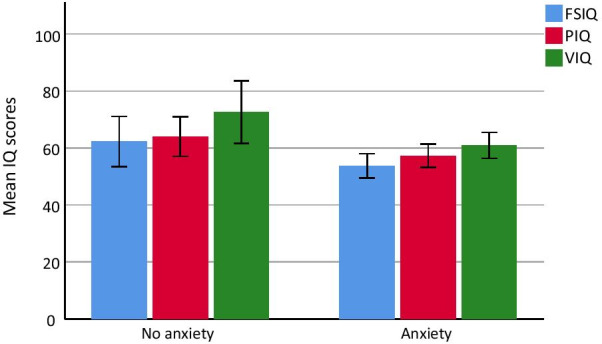
IQ scores split on anxiety vs no anxiety groups. Error bars represent 95% CI. There was a significant difference between the groups on all three variables (*p* < 0.05)

Descriptive information from the group comparisons is shown in Table 3 and Fig. 2. Whilst both groups showed a significant heterogeneity, the anxiety group had lower FSIQ (*t*(22) = 2.18, *p* = 0.020), as well as verbal (*t*(22) = 2.55, *p* = 0.009), and non-verbal IQ (*t*(22) = 1.90, *p* = 0.039), all one-tailed p-values. Although the other variables failed to show significant differences between the groups, it is notable that the anxiety group showed consistently worse performance on all measures, i.e. lower working memory scores, more parent-reported problems with EF, learning and memory, and worse performance on the CCPT. The Bayes factors are shown for the alternative hypothesis (i.e. lower IQ and worse executive functions in the anxiety group), and the null hypothesis (i.e. no difference between the groups). The Bayesian statistics gave positive evidence for differences between the groups for FSIQ; BF_10_ = 3.68 (i.e. the data is 3.68 times more likely to occur under the alternative than the null hypothesis), and verbal IQ; BF_10_ = 6.50 (i.e. the data is 6.50 times more likely to occur under the alternative hypothesis than the null hypothesis).

**Table 3:** Between-group comparisons

Variables	Anxiety (N = 15)		No anxiety (N = 9)		*t*(22)	Bayes factors	
	M (SD)	Range	M (SD)	Range	*t*/*Z*	BF_01_	BF_10_
Age^a^	27.6 (14.1)	9–53	32.2 (12.5)	16–49	0.83	2.04	0.49
CGI-S anxiety^a^	4.07 (1.03)	3–6	1.67 (0.5)	1–2	− 6.52***	< 0.001	> 500
FSIQ^a^	53.73 (7.66)	40–72	62.22 (11.50)	45–79	2.18*	0.27	3.68
Verbal IQ^a^	60.93 (8.22)	45–71	72.56 (14.26)	56–97	2.55**	0.35	6.50
Non-verbal IQ^a^	57.27 (7.32)	50–76	64.00 (9.0)	50–74	2.00*	0.15	2.87
WM^a^	64.73 (7.60)	51–79	67.56 (10.86)	53–82	0.75	1.46	0.68
FTF EF^a^	0.89 (0.34)	0.44–1.64	0.88 (0.51)	0.08–1.72	− 0.09	2.47	0.40
FTF Memory^a^	0.78 (0.31)	0.36–1.27	0.64 (0.37)	0–1.09	− 1.04	1.10	0.91
FTF Learning^b^	1.38 (0.38)	0.70–1.85	1.30 (0.61)	0.48–2.00	− 0.30	1.62	0.69
CCPT CR^b^	87.69 (11.70)	57–99	93.88 (3.94)	86–98	− 1.02	1.49	0.58
CCPT CE^a^	62.60 (18.38)	44–93	64.33 (16.37)	49–86	0.23	3.03	0.33
CCPT Variability^a^	178.70 (94.72)	48–385	112.12 (84.50)	40–304	− 1.67	0.56	1.77

BF_01_ = Bayes Factor for the null hypothesis, BF_10_ = Bayes Factor for the alternative hypothesis, CGI-S = Clinical Global Impression Scale—Severity, FSIQ = Full scale IQ, WM = Working Memory, FTF = Five to

*p < 0.05, **p < 0.01, ***p < 0.001

^a^Students t test was used, ^b^Mann–Whitney U test was used

## Discussion

We examined the role of EF for anxiety in the WS phenotype, and in contrast to our hypothesis, we found no association between anxiety and core elements of EF such as working memory, sustained attention, and inhibition. Neither did we find any association between anxiety and related variables, such as memory and learning. Rather, using Bayesian statistics we showed substantial evidence that there are indeed no associations between anxiety and inhibition, and between anxiety and learning, in our data. Using ordinal logistic regression analyses, we showed that younger individuals with more learning challenges are those most at risk of anxiety. The results for IQ and anxiety were confirmed in a group comparison between participants with and without diagnosed anxiety disorders. Finally, we analysed VIQ and PIQ separately and found that the association hold for both variables. In summary, the present study is novel in combining direct assessments, participants of a wide age range, and Bayesian statistics, and it highlights the importance of IQ and age as predictors of anxiety in WS. Below, we will discuss these results in relation to previous literature.

Our results partly replicate the findings by Pitts and colleagues [23]. They found that lower IQ and age increased the risk of receiving a specific phobia diagnose in WS. However, other studies have come to contradictory conclusions. In a large WS sample (n = 119), the general intellectual functioning, as assessed by the Differential Ability Scale, in children with and without a specific phobia diagnose were compared. No significant difference between the groups was reported [25]. Using multilevel modelling (n = 40), Woodruff-Borden and colleagues reported similar results, and no associations between IQ and anxiety disorders [16]. Methodological differences might partly explain the contrasting results in these studies in comparison to the present. The previous studies included children only, whilst the current study is based on a wider age range including adolescents and adults. Further, Leyfer and colleagues reported IQ within the normal range in 20% of their study sample [25], and Woodruff-Borden and colleagues reported a mean FSIQ of 78.2–79.1 in their sample [16]. In comparison, 92% of participants met criteria for an ID diagnosis in our sample and the mean FSIQ was 57. Hence, the relatively high proportion of intellectual disability in the current study, is another potential factor that might explain the study contrasts. In future research it would be relevant to examine whether the relationship between IQ and anxiety in WS is stronger in individuals with intellectual disabilities, in comparison to those with an IQ within the normal range.

Intellectual disability is in general associated with increased risk of anxiety disorders, in comparison to the general populations [13]. Still, studies comparing different severity levels of intellectual disability and its association with anxiety, are scarce. In a population-based study including all individuals with intellectual disability in Glasgow (n = 631), the authors reported that moderate, rather than mild intellectual disability, was associated with an increased risk of mental illness including anxiety disorders [51]. Our results of individuals with intellectual disability (or borderline intellectual disability) and WS, show that IQ plays a small, but robust and consistent role for anxiety in this population.

In addition to the effect of IQ and age, Pitts and colleagues found that parent-rated behaviour regulation problems were the strongest predictor of a specific phobia diagnose, when controlling for the effect of gender, age and IQ [23]. In contrast to these results, we show substantial evidence for no association between anxiety and inhibition in our data. Again, methodological differences might explain the study contrasts. Previous studies are based on parent-reports of EF (e.g. [15]), while the current study also measured inhibition directly by psychological testing. Toplak and colleagues reviewed 20 studies measuring executive functions with both performance based test and ratings, and reported that a majority of the studies (76%) showed no significant correlation between the measures [52]. They concluded that there are different underlying constructs captured in performance based tests and ratings; “Performance-based measures of executive function provide important information regarding efficiency of processing, but ratings of executive function tell us more about success in rational goal pursuit” [52]. We argue that both levels of executive functions are relevant in studies of behavioural phenotypes, but importantly, they are not interchangeable. Hence, more studies using direct measures of emotional regulation and anxiety in samples of WS is warranted for a deeper understanding of the mechanisms under-pinning anxiety.

In this study, some limitations need to be considered. The results from the regression analysis might be limited by the small sample size. In our data, we show a collective effect of IQ and age on anxiety, but fail to show that IQ and age are unique predictors of anxiety. These non-significant results might be due to a lack of power, and future studies with larger sample sizes are needed to further disentangle the role of age and IQ in anxiety in WS.

The measures of anxiety included in the present study, i.e. the MINI interview in combination with the CGI-S scale, are general standardised clinical measures. As such, there is a risk that these instruments fail to capture all aspects of anxiety in a population with atypical neurodevelopment such as WS. In a recent study by Royston et al., a novel approach to assess anxiety in WS was taken [53]. Parents were assessed with a semi-structured instrument created to explore the nature and phenomenology of anxiety in WS. The results from the study elucidate multiples triggers of anxiety, confirming previous results as well as novel findings (i.e. negative emotions in others), and highlight the role of anxiety management strategies. However, in contrast to previous studies, the results from the Royston-study show a low impact of anxiety on the individuals’ lives. The authors propose that among other factors explaining these results, one might be that parents underreport the extent of anxiety problems in comparison to the individuals themselves. In line with Royston and colleagues [53], we argue that studies of anxiety in WS using self-assessments and methods such as formulation interview techniques, or scales with good psychometric properties particularly developed for populations with intellectual disability, such as the Glasgow Anxiety scale or the Anxiety, Depression and Mood Scale [54], are important areas for future research.

Further, executive functions is an umbrella term for abilities, including working memory, attention and inhibition (investigated in the current study), but also abilities such as mental flexibility, problem solving, and planning. Future studies based on more comprehensive neuropsychological test batteries for assessment of executive functions (e.g. Delis-Kaplan Executive Function System), might be another important area for future research related to anxiety in WS.

## Conclusion

As has been shown by the literature, intolerance of uncertainty is probably the most prominent factor causing the high levels of anxiety in WS individuals [29, 30]. We add to that knowledge by showing that low intellectual ability increases the risk of having high levels of anxiety in WS, just as in other populations. Further, using Bayesian statistics, our results give evidence that behaviour inhibition is not associated with increased anxiety in WS. Together, these data illustrate a need of more studies based on direct measures combined with alternative statistical methods, for additional insights to the behavioural phenotypes of rare genetic disorders.

## Data Availability

The datasets used and/or analysed during the current study are available from the corresponding author on reasonable request.

## Abbreviations

WS: Williams syndrome
EF: Executive functions
ADHD: Attention-deficits/hyperactivity disorder
IQ: Intelligence quotient
SD: Standard deviation
DSM-5: Diagnostic and Statistical Manual of Mental Disorders 5th edition
CGI-S: Clinical Global Impression Scale – Severity
WISC-V: Wechsler Intelligence Scale for Children 5th edition
WAIS-IV: Wechsler Adult Intelligence Scale 4th edition
FSIQ: Full scale IQ
VIQ: Verbal IQ
PIQ: Performance/non-verbal IQ
FTF: Five to Fifteen questionnaire
CCPT: Conners’s Continuous Performance Test
K-CPT-2: Conner’s Kiddie Continuous Performance Test 2nd Edition
CPT-3: Conners’s Continuous Performance Test 3rd edition
WMI: Working memory index
BF_10_: Bayes factor in favour of the alternative hypothesis
BF_01_: Bayes factor in favour of the null hypothesis
CR: Correct responses
CE: Commission errors
ID: Intellectual disability
